# Domestic

**Published:** 1841-05-01

**Authors:** 


### DOMESTIC.

Contrary to our usual custom, we extract a paper, entire, on the milk sickness of the West. It was presented as an inaugural essay by the author, who had, as will be seen, considerable experience in the treatment of the disorder. It has long been desirable to obtain some accurate information as to the causes and nature of this affection, which is of so fatal and intractable a nature. The paper before us does not give us the key to its causes; perhaps they will never be discovered; but it is at any rate highly desirable that every effort should be made to develope the history of the affection, and, if possible, to ascertain some more favourable method of treatment than has yet been suggested.

*On the Milk Sickness of the West.* By GEO. B. GRAFF, M. D., of Edgar county, Illinois.—Occurring, as the milk sickness universally does in our frontier settlements, where medicine as a science is in its infancy, and its practice too much in the hands of the ignorant, we have had very imperfect accounts of the affection, either as regards its history, symptoms, or means of cure.

A few imperfect essays recorded in our medical journals, constitute every thing that I have ever seen offered to the public on this subject. The earliest history of it that I have noticed, is mention made of a singular disease affecting cattle, by Bishop Hennepin, a French missionary, who ascended the western rivers early in the last century. He knew of it only as causing the death of cattle with singular and often very violent symptoms.

The only name by which it is known, is that which I have used, which is quite objectionable, as it may serve to convey an erroneous impression by the supposition that milk only could produce it; whereas, the flesh of an infected animal acts with an equal degree of violence and rapidity. It is greatly to be desired that we should have for it a name which would be more appropriate, drawn either from the localities in which the disease is found, or from some distinctive character of the affection.

It is a disease peculiar to the United States, occurring seldom, if ever, to the eastward of the Alleghany mountains. It is in a greater or less degree met with in all the western states, as far south as Mississippi, and extends north to the boundary. The states of Indiana and Illinois are most subject to its occurrence, whilst its existence in the bordering states is comparatively rare. As I have already stated, from the want of medical intelligence and enterprise in these sections, the knowledge which we possess of its early history has been obtained only as a portion of the history of those neighbourhoods in which it presented itself; and in those instances it would have passed into forgetfulness, but for the dreadful ravages it committed in the diminution of the numbers of the early settlers. In the first formation of our western settlements, its prevalence often served as a cause to disband a community, and compel the inhabitants to seek a location which enjoyed immunity from its occurrence. Many of the otherwise most desirable portions of that country remained long exempted from settlement, and even now the inhabitants of these localities have, as a condition of their residence, entirely to abstain from the use of milk, its preparations, and the flesh of their cattle.

Its occurrence or prevalence is confined to no season, or description of weather, existing in a like degree in the heat of summer or cold of winter, and with like virulence and frequency during a dry or wet season. An opinion is entertained by some, that it is more frequently met with in the spring and fall months, whilst others have expressed a belief of its more common occurrence during the heat of summer. However this may be, we know of no season during which it does not occur.

The animals in which it has been observed are the beef cattle, horses, sheep, and goats, which seem to acquire it with their food or drink.

We will first speak of the *symptoms* manifested in cattle affected with it, as it is only through them that we have yet found the disease communicated to man. They may be affected to such a degree as that their flesh and milk will produce the disease, and yet they themselves manifest no unhealthy symptoms whatever. This latent condition of the disease may be discovered by subjecting the suspected animal to a violent degree of exercise, when, according to the intensity of the existing cause, it will be seized with tremors, spasms, convulsions, or even death. This is a precaution practised by butchers in these countries, always before slaughtering an animal in anywise suspected of the poisonous contamination. An ordinary degree of exertion will not develope these phenomena unless it produce the symptoms usually preceding a fatal termination. When, for instance, a cow is sufficiently deeply affected, nothing peculiar is observed until immediately preceding the outbreak of the fatal symptoms. She is then observed to walk about, without any apparent object in view; all food is refused, and there is evidence of impaired vision. The eye is first of a fiery appearance, increasing to a deepened red colour, until the animal is observed to stagger and fall, when, if she rise, the trembling of the whole muscular system will prevent the maintenance of the standing position. The animal usually dies after repeated convulsions, never lingering beyond a few hours. Often it falls suddenly, as if it received a blow from a heavy body on the head, and death is produced in a few minutes.

From the tremulous motion imparted to the muscles, the affection has received the common name of the "Trembles" in cattle. A case which was characterized by the great violence of its symptoms, I had an opportunity to examine very shortly after death. The brain I found suffused with a large quantity of fluid blood, which, from the amount contained within the cranium, must have made great pressure on every part. Unacquainted as I was with the healthy appearances of the other tissues, I could not speak of any changes existing in their appearance or structure.

In man the symptoms differ from these, and are varied. The length of time found to elapse from the reception of the cause to the appearance of the disease, is dependent on a multiplicity of circumstances, as the age, sex, or condition of the patient, and violence of the poison. It may be developed early as the third, or deferred until the tenth day. As a premonitory symptom, a peculiar and indescribable fœtor from the lungs is the most prominent, and so universally have I found it present and to precede the disease, that in almost every instance where I have been brought in proximity to a person predisposed or attacked, have I been able to foretell its approach, and pronounce on the character of the disease. This fœtor can no more be mistaken by a person accustomed to it, than that which is so universally attendant on variola; and it may in fact be safely stated to be pathognomonic of the forming and early stage of milk sickness. This halitus from the lungs, which I have never found entirely wanting even some days previous to an attack, increases in intensity until the disease is fully developed, when it gradually disappears with the specific symptoms, and at the termination of four or five days cannot be detected. A person labouring under the peculiar effluvia from the air passages, in many cases complains of no illness, and appears entirely unconscious of his situation, unless advised of it by his friends or attendants. His appetite may be, and usually is, destroyed; and after the lapse of a few days he is taken down with pain and excessive irritability of the stomach, obstinate constipation of the bowels, a cessation of all biliary secretion, general febrile action, sometimes an intense burning sensation in the epigastric region, with early and obstinate coldness of the extremities. Often the symptoms are observed to differ widely from these. Besides the peculiar smell emitted, there is a premonition of the attack; for some days previous to its development, the patient experiences a restlessness and uneasiness which he cannot describe; there is a frequent moving about without any definite object in view, and he finds it impossible to confine his attention to any subject or employment. He feels, and often expresses a dread of some impending calamity; starts at the slightest noises; his temper is always irritable; his lip is seen to quiver when he attempts to speak, and all his motions are characterized by a nervousness, and are quickly performed. This state of things gradually increases in severity; his ideas are much confused; he suffers greatly from a want of words to express his meaning, with every evidence of a deep and somewhat peculiar state of cerebral irritation. Added to these there is a severe pain in the head, attended with tinnitus aurium, suffusion of the eyes, and intolerance of light. Vomiting announces the onset of an attack, and consists at first only of the contents of the stomach, which are violently ejected. Some bile may or may not be discharged; it is for the most part absent, and in some instances I have seen large quantities of dark coloured and viscid bilious matter thrown up; but it continued for only a short time, when it ceased, and all evidences of its secretion are absent until it be restored by the action of remedies. Vomiting at short intervals continues for many days, consisting of the fluids swallowed, mixed with a glairy mucus, and not unfrequently tinged with blood. Some days frequently elapse before pain in the stomach is complained of; but during the time the suffering is intolerable, consisting of a sensation of deep distress, which, though referred to the præcordia, or abdomen, the sufferer cannot locate in any particular spot. Pain in the limbs is complained of, and is severally referred to each of the extremities, but is more constantly located in the spine, particularly at the nape of the neck. The pulse, during the forming stage, possesses greater force and volume, with slightly increased action. Upon the commencement of vomiting, it becomes greatly accelerated, is quick and frequent, and varies in different cases with the degree of inflammatory action existing, and the means of treatment employed. The bowels will remain obstinately constipated, the powers of nature being incompetent to relieve the condition, so that unless it be done by appropriate remedies, at the end of six or eight days an offensive discharge takes place, quickly followed by dissolution, the symptoms being those which would indicate disorganization of the structure of the intestines. The tongue, during the initiatory stage, is slightly furred, but otherwise not much changed in appearance. This coat disappears soon after the occurrence of vomiting, and becomes clean, of a pale red or pink colour, greatly resembling a piece of raw veal. Next to the fœtor mentioned, the change of volume occurring in the tongue may be viewed as the great characteristic of this disease. It rapidly attains an inordinate size, completely filling the mouth, and so flabby and soft in its texture as to retain perfectly the impressions left by the teeth when extruded. Often a number of efforts are necessary before it can be forced out, and then it has a tremulous motion. This condition of the tongue changes with the stage of the disease. When the vomiting has been suspended, and free evacuations from the bowels obtained, it is reduced in volume, the surface is for a time smooth and glazed, soon after becomes dark, cracks open in transverse fissures, is hardened with an obstinately dry and rough surface. Of all the primary symptoms, vomiting is the last to disappear; it ceases very gradually to annoy the patient, and its continued absence is the most certain indication of a state of convalescence.' In no disease is there a greater difference or diversity of symptoms than are usually found in different cases to constitute what may be properly termed the secondary stage of milk sickness.

The economy at large seems to suffer in proportion to the intensity of the vascular and cerebral excitement, with the local direction impressed on them, as well as from the remedial means found necessary to be employed during the course of treatment. Simultaneous with the disappearance of the peculiar odour emitted from the lungs, the disease loses all its specific characters, and the system seems to labour to recover from the injury inflicted upon it. Often the symptoms are those evincing only extreme debility of the powers of animal life, with general functional disturbance. In other cases the patient is affected with drowsiness, low muttering delirium, nervous tremors, and that whole train of cerebral disturbances which indicate a low typhoid condition of fever. This latter condition occasionally follows all modes of treatment, but is particularly liable to present itself when a due degree of depletion has been neglected, or the efforts to evacuate the bowels have not been sufficiently vigorous, and will most readily happen during the winter, and early in the spring. In this stage the skin is hot and dry, or cool and moistened, with a clammy and particularly offensive secretion. The coldness of the extremities will be found to remain until the condition of, and disposition to congestion, is entirely broken up. The degree of sensibility of the bowels on pressure is extremely variable; sometimes an application of it in a moderate degree being attended with pain, and at others none at all. The volume of the bowels is increased, and they have externally a soft doughy feel, and one of the most common appearances is a regular pulsation of the entire abdomen, seeming to be greatest at a point to the right of the umbilicus.

Frequent fits of extreme restlessness occur which cause much suffering, the distress increasing until entirely relieved by the vomiting up a few ounces of a dark coloured fluid, much resembling coffee grounds; after which the patient soon relapses into his former state of stupor or insensibility to all surrounding objects. When this latter symptom has been present, I have never witnessed a case of recovery. It seems to occur in these instances in which the liver was primarily deeply engorged, and I have not observed it when the biliary secretion had been completely established. According as the establishment of the secretion has been early or retarded, are the chances of recovery, and mildness of secondary symptoms in a direct proportion. When recovery does ensue from severe cases of this affection, it is tedious, and years are often required to restore the patient to his wonted health and vigour. Indeed it has been a question with many, whether those once severely attacked ever regain a perfect integrity of constitution. In cases which terminate fatally, (of which description is a large majority,) a length of time of from one to four weeks is required, proportionate to the intensity of the primary effects, the propriety of the treatment, and the natural powers of the resistance of the constitution, as they often seem to die from a wearing out, or gradual destruction of cerebral and nervous energy. Those cases which occur during the summer months, are most decidedly inflammatory, whilst in the winter there is always observed a disposition to assume a low form. The autumnal cases, in their secondary fever, are liable to assume a remittent aspect, and I have seen them eventuate in a well marked intermittent. When recovery has taken place, the patient retains not the slightest recollection of any thing which occurred during the progress of the disease, and this forgetfulness often extends as far back as some days previous to the active development of the disease. In a single instance, and the only one of which I have ever heard, I witnessed incurable insanity to follow an attack of milk sickness, although the case was throughout attended with unusually mild symptoms.

The first evacuations obtained are watery, small in quantity, and almost without smell. When bile begins to be discharged, their colour changes from a light appearance to a green or darker one; they become consistent, and extremely and insufferably offensive. The quantity of the secretion of urine is throughout diminished, and at times almost suspended. At first it is observed to be high coloured, and deposits a very copious precipitate of acicular pink crystals, whilst in the more advanced periods of the disease it is colourless, and on standing very soon precipitates a quantity of mucus, without a change of colour. Nothing can be more widely different than the appearances of the blood drawn at different stages of the disorder. Taken at the onset of the attack, it is found dark, thick, and rapidly coagulates, presenting the buffed coat, well marked; the coagulum likewise contracts, and is deeply cupped. The quantity of serum is proportionately small, is invariably of a bright yellow, or even orange colour. This character is maintained, though in a degree less marked, for two or three days, when it greatly changes. The serum is found to predominate, is of a red colour from the red globules, which do not separate from it; the coagulum is quite small; long in separating; may be easily broken up, and presents a brown, gelatinous appearance.

*Cause.*—The cause of this disease in animals is as yet shrouded in mystery and uncertainty. No satisfactory account of its nature has ever yet been given, and it has in turn been supposed to be of vegetable, mineral, and even aërial origin. The limits of its prevalence is not often over a large continuous tract of country, but rather circumscribed, and surrounded by localities never known to produce it. No example is known in which the property of producing the disease has been acquired by any locality which did not previously possess it. The boundaries which were at the first discovery of the country found to separate the infected from healthy districts, remain unchanged. The locality which serves to produce the disease, most commonly extends as a vein of variable breadth, traversing the country for a considerable distance. It can be traced in one instance for nearly a hundred miles, running parallel to the course of the Wabash river, in the state of Indiana.

Again—it will be found to occupy an isolated spot, comprised in an area of one hundred acres, whilst, for a considerable distance around, it is not produced. Thus having the locality perfectly circumscribed, much labour has been expended in order to discover some production peculiar to the locality. The search has been uniformly unsuccessful in the attainment of its object. The general appearance of these infected districts is somewhat peculiar. I have always observed that the situation of the ground is elevated above that of the surrounding country, occupying what is denominated a ridge, and that the quality of the soil is in general of an inferior description. The growth of timber is not observed to be so luxuriant as in situations otherwise similar, but is scrubby, and stunted in its perfect development, in many instances simulating what in the west is denominated "Barrens." Throughout the entire district in which these localities are interspersed, there is observed an absence of the occurrence of stones scattered over the surface, whilst in the infected tracts they are almost universally present. They are of a small size and darkened aspect externally, breaking with a regular and shining fracture, and, upon analysis, imperfectly made, were found to contain a considerable portion of iron, with slight traces of copper. Another more decided and peculiar appearance which serves to distinguish them from other spots, is the breaking forth of numerous feeble springs, furnishing but a trifling supply of water, but not varying in quantity with the change of seasons. In its appearance, it presents the general evidences of a sulphurous and ferruginous contamination.

Experiments made upon the water collected from these springs, or more properly called oozes from the soil, with the greatest care by the employment of the most delicate chemical re-agents, failed to indicate the presence of any mineral except iron, sulphur, traces of magnesia, and a quantity of copper barely capable of being demonstrated. A belief being entertained by many that the disease is occasioned by arsenic, or some of its salts, I with much care and patience subjected not only the water, but likewise the earth from these districts, to a most rigid examination, and by no test was I furnished with the slightest evidence of its presence.

An intelligent medical friend expressed to me his belief that it was produced by the inhalation of some noxious gases generated during the night; in proof, he stated that he had observed cattle, which were regularly housed each evening, escaped its attacks, and that when suffered to remain at large, they were frequently seized with the disease. It is difficult to form this belief of the nature of the cause, as we can hardly conceive the particular action of any combination of circumstances, capable of giving rise to such an emanation only at night, ceasing to act during the day. The most popular belief is in favour of a vegetable origin. The advocates of this method of production having failed to designate the plant which they supposed occasions it, have endeavoured to sustain their views by supposing that the poison exists in some shrub or tree, which is eaten by the cattle, but confess their inability to designate any such peculiar growth confined to these localities. If certain fields which are known to affect cattle fed upon them, be suffered to grow in grass, and the hay produced be given to them for their continual food, no disease results, which is a strong circumstance, unless it be urged that the active poisonous principle is destroyed by the desiccation. Again, it has frequently appeared with its greatest virulence when the ground has been for weeks previously covered with snow.

For my own part, I would most willingly subscribe to the opinion that some mineral, or mineral combination possesses the agency of its production. Yet I confess that I cannot even imagine what must be the nature of that substance producing such violent and anomalous effects, and in its operation so unlike any thing with which we are acquainted. The cause, whatever it may be, when it enters the organization of the animal, either by inducing a specific action in the tissues of the economy, or by a combination with some of the elements of the body, forms a poison not more violent in its operation than singular in the effects it can produce. If this cause should prove to be a mineral, it must be one of great subtlety from its difficulty of detection, and from its virulence it must possess qualities and activity not equalled or resembled by any metal or metallic combination yet discovered. No substance, of which we possess any knowledge, will produce like phenomena. Hoping, that if I could succeed in developing the same symptoms and effects by some active or poisonous article, it might, by the probable analogy of the agents, lead to the discovery of the nature of this poison, I patiently tried many. The action of none of the mineral poisons were found at all similar. My experiments were chiefly made on dogs, and in them I found the symptoms, immediately preceding their death, occasioned by a fatal dose of strychnia, greatly to resemble those produced by the continued admininistration of the flesh of an animal which had perished from milk sickness. The appearances, on dissection, differ in a greater degree, and particularly in that case of poisoning by the vegetable proximate principle exhibit the blood in a state more nearly resembling a healthy condition. With the view to an extensive series of experiments, I procured the body of a full grown cow, which had perished suddenly from the affection, with violent symptoms. The brain was immersed in a copious effusion of blood, and in no portion of the body was it found coagulated. The flesh, in external appearances, did not differ from that of healthy beef, unless that it was slightly darker, and a thin bloody fluid continually dropped from it. By exposing it by the side of a healthy portion, I found that the influence of the sun rendered the specimen from the diseased animal offensive, and turned it to a greenish hue, whilst the other remained comparatively sound and unaffected. It can possess nothing peculiar in its taste, for persons who have partaken of it have not remarked any thing unusual, and animals will exercise no preference, if the two descriptions be simultaneously presented to them. The beef which I procured was subjected to the ordinary process of salting, which did not in the least affect its poisonous properties.

Butter and cheese, manufactured from the milk drawn from an infected cow, are supposed to be the most concentrated forms of this poison. They possess no distinguishing appearance, odour, or taste, from the healthy article. A very minute quantity of either will suffice to develope the disease in man. The cream, ordinarily sufficient to be added to the coffee drunk at a single meal, is said to have induced an attack. The butter or cheese eaten at one repast, has frequently been known to prove effective. The property is not contained in any of the elements of the milk exclusively, but distributed throughout the whole of them, being possessed by the buttermilk as well as by the whey. Beef, in the quantity of a very few ounces, will produce the disease, and it is generally believed in a more violent and fatal form than when it is produced by milk, or any of its preparations.

In the course of my observations I had an opportunity to experiment with a cow suffering in but a slight degree from the cause. She was affected with tremors when unusually exercised, exhibited a red and suffused eye, with frequent twitches of portions of the muscular system. She was kept confined without an opportunity to exercise, and was fed upon ordinary food. At the end of eight days, the milk drawn from her possessed as violent poisonous properties as at the time of her incarceration. Her confinement was continued for a week longer, at the end of which period, the milk taken from her was found in an entirely healthy condition, and the eyes were restored to their natural appearance. In this instance it will be seen that the property of imparting the poison to the milk was lost in the space between eight and fifteen days. We, of course, cannot fix on the precise period, but we would infer that the property is suddenly destroyed rather than gradually dissipated.

My trials with the poisoned flesh were, for the most part, made upon dogs, which I confined, and often watched the effect of the poison when administered at regular intervals. In the space of forty-eight hours from the commencement of the administration of either the butter, cheese, or flesh from poisoned animals, I have observed unequivocal appearances of their peculiar action. In a few hours, a thirst greater than natural is created, the appetite remains unimpaired until the expiration of the fourth or fifth day, or just before the appearance of fatal symptoms, the animal will refuse drinks and the most inviting descriptions of food.

Vomiting does not, as in man, always precede death, but the bowels are constipated throughout, except that, in a single instance, I observed copious alvine discharges largely mixed with blood. One ounce of butter or cheese, or four ounces of beef, either raw or boiled, administered three times a-day, will certainly prove fatal within six days, and often earlier. In these cases, all exertions and exercise must be prevented, or death will occur much sooner, even as early as the third day. When an animal has been subjected to its influence for only a short time, and is induced to fatigue itself, or is driven a distance at full speed, he suddenly stops and falls, and the severity and duration of the convulsion or spasm, is in proportion to the intensity of the action of the poison. Often he will appear to entirely recover from the first attack, but to be repeated upon the renewal of the exercise to a sufficient degree. With a dog in this situation, I endeavoured to keep him under a moderate degree of its influence, giving the flesh in very small quantities. Soon his stomach became so irritable that it rejected all solid food swallowed, but retained water, which he drunk in large quantities. In this state he lingered along, rapidly emaciating, taking no exercise unless compelled to move; he slept nearly the whole time, seemed lethargic, and lost entirely the remembrance of his former master. He died without a convulsion, and I instituted an examination of the post-mortem appearances, which may be found in the relation of Case II. Of the symptoms produced, I have observed a great diversity, but their difference can be accounted for by the amount of the poison administered, and, perhaps, the different degrees of susceptibility of the various animals experimented upon. One of the animals, which I proposed to destroy with large doses frequently repeated, escaped from confinement during the second day, and I saw him enjoying apparently excellent health some weeks afterwards. The effect of the poison must be manifested throughout the entire system, and vitiate all the secretions. An experiment, which went far to prove how deeply the milk of other animals is imbued with its poison, was made by administering the infected meat to a bitch suckling five puppies. The effect produced in them was very sudden, and the entire litter died in four days, which was two days before the occurrence of the death of the mother. I am inclined to believe that the poisonous principle exists in a more powerful degree in the muscular fibre, than in either the cellular or adipose tissue. I could observe great difference in the violence of the effects, when the same quantities by weight, of either of these, was administered.

The urine secreted by man during the first or inflammatory stage, is highly charged with the poison. It is secreted in such a diminished quantity that it could not be sufficiently tested: but by evaporating the small quantity obtained to the consistence of honey, I have succeeded in producing well marked symptoms, though in a slight degree.

That the flesh of a man dying with this disease, should, if eaten, communicate it, we can believe, as that of the dogs destroyed possessed it in as high a degree as the beef which first communicated it. In order to discover, if possible, whether the active principle may not prove to be some matter, the constitution of which might be determined by subjecting the poisonous flesh to chemical agents, numerous experiments were instituted having this object in view. In one instance, a quantity of the flesh was cut into small pieces, and subjected to the action of dilute sulphuric acid for two hours. It was then thoroughly washed with repeated quantities of cold water, and given to a dog; it seemed to have parted with none of its active properties. The same experiment was made with the other mineral acids, likewise with the tartaric, and with the like results. In succession, I treated the muscular tissues (as being the portion most actively poisonous,) with the chlorides of lime and soda, the alkalies and tepid water, without apparently modifying the effect of the poisonous principle in the animal system. Heat, when conjoined, did not affect the result. I found one agent, and one only, which seemed to exercise the least perceptible action upon it. When the flesh was boiled for a length of time in a decoction of Aleppo galls, and afterwards carefully washed with water, I found it to be rendered comparatively innoxious; but even then a large quantity of it administered would produce slight manifestations of the peculiar effects of the poison. With this experiment to guide me in its treatment, I administered tannin repeatedly, and in large doses at the onset of the disease, but with such a result as convinced me of its doubtful efficacy.

Butter of a poisonous quality, subjected to such a degree of heat as to cause it to inflame, loses none of its virulent properties. With a view to ascertain if the poisonous principle was soluble or could be communicated to water, I boiled a large quantity of the beef in pure water for several hours, and afterwards evaporated the liquid thus obtained to the consistence of cream. Although this extract contained a large quantity of gelatinous matter, with some of the other constituents of the flesh, yet, on being given in large quantities, no perceptible effect was produced. From this we can draw the practical inference that this disease will not be communicated to man, even should he partake of soup made from an infected portion of beef. As far as my researches have extended, I have been unable to produce the disease by an inoculation with any portion of the body, or secretions from infected animals. If we reason from analogy, we might incline to the opinion that some product of the diseased action would possess the power to reproduce it, and suppose it to exist in a concentrated form, as we have in the saliva of hydrophobia, or the matter of variola.

That a matter possessing like activity may exist in some part not yet demonstrated, we can readily suppose, but we must wait for patient investigation to determine the truth or fallacy of the supposition. This subtle poisonous principle, of whatever it may be proved to consist, seems to possess the power of infinite reproduction, by some vital or chemico-vital action of the system of those animals poisoned by its influence. Thus, suppose one pound of flesh to prove sufficient to produce the death of another animal, it will be found that each pound of flesh of that animal so destroyed, will possess as active powers of destruction, and will, in its turn, serve to contaminate the whole body of another animal in the same degree. I had wished to repeat this so often as to demonstrate the possibility of its reproduction *ad infinitum*. In this I failed, from the difficulty experienced in compelling "dog to eat dog." From what I have observed, I would say that there exists a power of generation of the poisonous principle in the animal economy. This primary impulse being received from the specific action of some substance, vegetable or mineral, by some means obtained from the soil.

There is, however, one animal, which, from some peculiarity of organization, is rendered proof against the pernicious effects of this otherwise powerful agent. I allude to the hog. Most industriously did I feed a troublesome sow running at large, administering daily five or six pounds of infected beef. This was persevered in for more than a fortnight, and under the *treatment* she fattened, when I was compelled to desist, from the great quantity necessary to supply her voracious appetite, without enjoying the satisfaction to perceive one muscular twitch as an evidence that it produced the slightest effect. When I last saw her, she enjoyed excellent apparent health, and was the mother of a numerous offspring.

Of the appearances presented on *autopsic* examination, I shall first speak as they were found in animals. For the sake of brevity, I shall notice only two cases, selected from a number made and recorded. The first is that of a young and healthy dog, which was fed on large quantities of poisoned meat regularly for three days, during which time he was kept perfectly quiet. At the end of this period he was taken out, and run violently a few hundred yards, when he was suddenly seized with a severe convulsion, and died in a few minutes, not having recovered sufficiently to be able again to rise to his feet. The examination was made one hour after.

The external appearance of the stomach and viscera of the abdomen was healthy, except that the intestines were much diminished in their calibre, and the former organ was reduced at least to two-thirds of its natural size. The mucous lining was of very pale red, or pink blush, and the appearance extended over every part. Blood in a considerable quantity was found upon the surface, and within the ventricles of the brain. The veins were distended with a dark liquid blood, and in no part was it found coagulated. The arachnoid membrane, when washed from the blood effused around it, was found to be very minutely injected, and in places rendered slightly opaque. No examination of the spinal cord was made. This will serve to give a general idea of the appearances in acute fatal cases. Of a number examined, no one differed essentially from what we have described. When the medulla spinalis has been exposed, it gave evidences of recent irritation, and there was likewise a general phlogosed appearance of the liver, lungs, spleen, and kidneys.

The next examination to which I shall allude, is that of the case spoken of in page 284, having been one of long continuance. This examination was made three hours after death, when the abdomen being opened, was found to contain a quantity of bloody serum, and adhesions of a slight nature had been formed between the surfaces of the peritoneum. The appearance of the membranes indicated different degrees of inflammation in different portions, and in some spots it had assumed a dark and gangrenous aspect. The liver was of an extremely dark colour, distended with blood, and its texture was much softened. The spleen was more than double its natural size, and its substance was easily broken up. The stomach was contracted to the size of an orange, with no portion of the lining membrane in a sound condition, being softened, and for the most part disorganized, but in no place perforated. This altered appearance of the mucous membrane became gradually less marked, as the examination proceeded downwards, and the lining membrane of the large intestines was nearly natural in appearance. Upon exposing the brain by removing the dura mater, there was found beneath it a layer of coagulable lymph and purulent matter covering its entire surface. In some portions the tunica arachnoides was found opaque, and so much thickened as to have changed its physical character. Within the ventricles, patches of coagulated lymph were effused. The substance of the brain, particularly that of the anterior and middle lobes, was found much softer than in health. The cerebellum seemed to have been a principal seat of the inflammatory action, although it retained entirely a healthy consistence. The medulla spinalis was exposed completely, and examined with care. Its membranes bore all the evidences of recent inflammation, and the substance of the cord was found minutely injected. Throughout the whole body the blood remained fluid, and at no place was the smallest coagulum to be discovered.

From all the experiments I have made, and the reasoning used, I can arrive at no conclusion, so far as relates to the nature of the ultimate cause in man, to whom it can only be communicated through the medium of an animal, and that capability of production can be acquired only by the animals of circumscribed localities. An intelligent medical friend, alike distinguished as a statesman, Dr. John W. Davis, of Indiana, in a late letter to me, expresses a belief that milk is never a cause of the disease. He merely states his belief of the fact, without the evidences or observations which have led him to the denial of a proposition heretofore viewed as settled beyond dispute. My own experience enables me to say that I have seen a peculiar affection, which I feel assured could have been no other than the milk sickness, in a city remote from any of its local causes, attacking every individual who partook of a certain cheese which had been purchased from a wagon arriving from an infected district. In this instance, the well marked symptoms, confined to those only who partook of this cheese, appearing nearly at the same time, with no occurrence of new cases after the removal of this cause, all together afford strong evidence of the nature of the origin.

The instances just cited occurred in Chillicothe, Ohio, in which place I met with a medical friend who had charge of a number of the cases. He expressed to me his belief that the disease was milk sickness, from what he had learned in relation to it, in addition to the fact that it was confined exclusively to a single house, sparing those inmates who did not eat of the cheese in question. At his request I accompanied him to see two of them, and, from every appearance and symptom manifested, I could not for a moment hesitate to form an opinion as to the real character of the disease. Fortunately, the attacks were of unusual mildness, and but two cases out of thirteen proved fatal.

There is a murderous practice now carried on in certain districts, in which the inhabitants will not themselves consume the butter and cheese manufactured; but with little solicitude for the lives or health of others, they send it in large quantities, to be sold in the cities of the West, particularly Louisville, Ky., and St. Louis, Missouri. Of the truth of this I am well apprised by actual observation, and I am as certain that it has often caused death in those cities, when the medical attendants viewed it as some anomalous form of disease, not suspecting the means by which poison had been conveyed among them. Physicians of the latter city, having been questioned particularly on this subject, have mentioned to me a singular, and often fatal disease, which appeared in certain families, the cases occurring simultaneously, and all traces of it disappearing suddenly, and which I cannot doubt were the result of poisoned butter or cheese. This recklessness of human life it should be our endeavour to prevent, and the heartless wretches who practice it should be brought to suffer a punishment commensurate with the enormity of their crime. From the wide extent of the country in which it is carried on, we will readily perceive the difficulties to be encountered in the effort to put a stop to the practice. This being the case, our next proper aim should be, to investigate the nature of the cause, and establish a more proper plan of treatment by which it may be robbed of its terrors, and the present large proportionate mortality diminished.

*Pathology.*—Of the essential nature of the cause, its operation, and mode of treatment, each professional man has his own peculiar notions and belief. One who has had much experience in the disease, will recommend a remedy as proving in his hands eminently successful, which another, equally experienced, would assure us he found entirely to fail, or greatly aggravate the symptoms. Indeed, so much discrepancy is found to exist between the relations of different practitioners, that we can hardly believe the affection in their respective localities to be identical, were it not for the one continual concomitance of that peculiar odour from the lungs already dwelt upon, and which all recognise in the genuine affection. We may reconcile these discrepancies by the supposition that the differences can be accounted for in the variations of the intensity of the cause, and the modifications impressed upon it by locality, season, and climate.

Hundreds of persons throughout the West and South-west, are annually perishing from its attacks. Owing to the want of successs which has so uniformly attended the practice of their physicians, many of the inhabitants depend entirely on their domestic remedies. It is in that country emphatically one of the *opprobria medicorum.*

The primary operation of the poison seems to me to be on the brain and nervous system, and this is indicated by the cerebral irritation which so often precedes, and always accompanies, an attack, as well as by autopsic appearances. Without an exception, in the animals poisoned, I always found the brain and meninges phlogosed with a greater or less degree of inflammatory action.

A case which occurred in my own practice, I think, fully sustains the position taken, and settles the matter, that the first impingement is made upon the general nervous system. The circumstances of the case were as follows:—The entire family of a Mr. Frazier, moving westward, purchased a quantity of fresh beef in Indiana, of which every member of the company partook heartily, daily, until it was exhausted, which was the day on which they arrived in my neighbourhood, being the evening of the fourth day. In the evening they retired, apparently in their usual health; but during the night I was summoned to attend a female of the company, who was advanced about six months in pregnancy. She was violently seized with pains of labour, and was prematurely delivered of a healthy looking fœtus at the moment of my arrival, which was in less than an hour from the commencement of her pains. The abortion was characterized only by the violence of her throes, and consequent severe suffering. No cause whatever could be assigned for its occurrence, her appetite had been good, and health as usual, except a slight occasional palpitation of the heart, which she attributed to the fact that her bowels had remained constipated for two or three days previous. Not deeming my presence necessary, I returned home only to be summoned again before morning, when I found unequivocal symptoms of milk sickness had presented themselves. The peculiar odour was quite perceptible, and, probably from the fact that I did not suspect the existence of the disease at my first visit, I failed to detect it. Upon a careful examination, I discovered the smell present with every member of the family, and, on inquiry, ascertained about the beef and the locality in which they purchased it, which at once satisfied me they were doomed. Before the next morning, every member of that company of six was attacked in a violent manner, and only one of the number recovered.

The woman first attacked was the first victim, her death having been hastened by the supervention of a dark-coloured hæmorrhage from the uterus on the day of her death, which was the fourth of the attack. In this case, I obtained an opportunity to institute a post-mortem examination. The viscera of the abdomen all bore traces of irritation and a slight degree of inflammation. The mucous membranes of the alimentary track were reddened in places, and the size of the stomach and the calibre of the intestines were somewhat diminished. The uterus had not perfectly contracted, and its walls wanted their usual degree of firmness; its lining membrane was darkened, and the surface was covered with a muco-gelatinous exudation of a light brown colour. Effused into the cavity of the peritoneum were a few ounces of reddened serum, and the membrane was discoloured, especially at the portions reflected over the fundus of the uterus. These appearances would not account for so sudden a death, and, on examination, the principal lesions were discovered in the brain. Occupying the ventricles, serum suspending flakes of coagulated lymph were found in a considerable quantity. The dura mater adhered to the membranes beneath it, and was detached with some degree of difficulty. The pia mater had so plenteously effused lymph upon its surface that it could not be separated from the arachnoid, which latter membrane, in some places, had lost its distinctive appearances. The substance of the brain itself was decidedly softened, and, upon examining a section of the cineritious portion, small and innumerable vessels, deeply injected with red blood, could be distinguished with the naked eye. The appearance of the liver was somewhat peculiar; it was deeply engorged, although depletion had been practiced to its fullest extent; it was of a darker colour than natural, and the gall-bladder was distended with a large quantity of viscid bile. In connection with this latter appearance, it is proper to observe, that none of this secretion was vomited at the onset of the disease, nor had the remedies the effect to restore a secretion of it.

This account must necessarily be imperfect, from the fact that the examination was made under disadvantageous circumstances,—by stealth at night, in the open air, and by the light of a single candle, and one assistant present.

I removed the brain for future inspection and exhibition, but the fact having been ascertained, the inhabitants insisted on a return of it, probably for the use of the deceased, and evinced their holy horrors of the practice of making post-mortem examinations, by carefully guarding the repositories of the others who died of this disease, until long after decomposition had rendered an exhumation unprofitable.

Never having seen an account given of any autopsic appearances in fatal cases of this disease, it would have proved to me a source of peculiar gratification had I been permitted to have instituted examinations in the cases of the other members of this family who died. I feel an anxiety to know if these evidences of violent cerebral inflammation were the legitimate production of the disease, unconnected with the puerperal condition of this patient. I have never yet witnessed a case of this disease in which the brain and nervous system were not deeply, and often primarily affected, which condition alone enables me to account for the abortion in this case.

*Treatment.*—General bleeding is the first, and, according to my experience of all others, the most important remediate means. The extent to which it should properly be carried, can be regulated only by the pulse, and, so long as *it* will bear it, we should have no hesitation to continue the abstraction, being guided neither by the weight nor measure of the amount drawn. To insure its advantageous operation it must be practised early, as, after the lapse of a few days, it cannot be carried to any extent from the sinking induced. If it can be avoided, we should never at one time push it so far as to produce syncope, for reaction is always slow, often imperfect, and we have seen congestion occasionally result from a want of attention to this direction.

The pulse I have ever found to be the surest guide, but occasionally it will, in the very commencement, be found small, frequent, and oppressed, and but slightly tense from the beginning. In such cases it is proper to open a vein, and, if the pulse rise whilst the blood flows, it should be continued.

Next in importance to general blood-letting, I would rank local abstractions by cups, applied over the epigastrium and region of the liver, but more particularly are they of service to the temples and back of the neck. In some recent cases terminating favourably, I have attributed the recovery mainly to the adoption of the latter means.

We should direct the head to be shaved in the beginning, cold applications being made and continued for some days. This, conjoined with the liberal abstraction of blood generally and locally from the head with cups, will be found the most certain means of allaying the excessive irritability of the stomach. Blisters may be applied early, and are found particularly serviceable over the liver, in which case they seem to have the effect to relieve the congestion of the organ, and hasten the resumption of the exercise of the suspended function. In the latter stages they are of much advantage applied to the top of the sternum, back of the neck, or to the scalp itself, and their good effects are increased by dressing them with some irritating cerate, in order to maintain a discharge. In certain low forms of the secondary fever, they will assist the oppressed powers of life, when applied to the extremities, being careful to keep up a continued impression by their repetition.

Opium and its preparations, given with a view to relieve vomiting, instead of effecting the desired object, have a direct tendency to increase it, and at the same time greatly to aggravate the febrile and cerebral symptoms.

No remedy administered internally has ever yet been found to allay either the irritability of the stomach or that intense sense of suffering which accompanies it.

By some the object is said to have been attained by the administration of repeated doses of tartarized antimony in the quantity recommended to maintain a state of nausea without vomiting. I view the propriety of its administration as questionable, and if it be really true that it does relieve the vomiting, we would account for it by tracing the effect to its indirect action on the brain. No agent will, according to my observation and experience, prove of service to allay this condition of the stomach, unless its operation be directed towards the brain; and, hence, the next important indication to blood-letting, is the application of the remedies already spoken of to that organ. The coldness of the extremities is a troublesome symptom, and can be relieved temporarily by the application of dry frictions and stimulating liniments. The extremities will not regain a natural and equable temperature until the proper biliary secretion is fully established.

The irritability of the stomach being quelled so far as that it will retain medicines, we must push their use with all possible industry, to relieve the existing obstinate constipation, and likewise restore the suspended action of the liver. For this purpose, resort is commonly made to calomel in large and repeated doses, and, at the same time, conjoin with it other purgatives of the most active class. I would condemn the freedom and frequency with which this mercurial has been administered, and suggest a reduction of the quantity to that not exceeding five grains every two or three hours, and entirely discard the use of irritating or drastic cathartics. A remedy that, in domestic use, has acquired a high reputation in the treatment of milk sickness, is olive oil. I have found it to be more readily retained in the stomach than other cathartics, and, though not very speedy, is certain in its operation. Conjoined with, or preceded by, the administration of a small dose of calomel, I have in many instances found no other medicines of the class necessary.

Soon after the commencement of the use of cathartics, we should have recourse to stimulating enemata every few hours, as they frequently exert a powerful influence in hastening their operation. Even when we have succeed-in opening the bowels and establishing the flow of bile, the cure of the case is but half accomplished, and much danger yet exists of a fatal termination. The secondary fever will always follow, with a different degree of obstinacy, varying as much in duration as in the different types it assumes. Of course, the treatment of this must be varied according to the symptoms which arise. Not even general directions can be given; there is now no specific action, and the intelligent practitioner will treat the symptoms as they present themselves.

I have now brought to a close a hasty and imperfect sketch of a disease which must be viewed almost as an anomaly in medical science. Our knowledge of it is unfortunately so limited, that we know not where to class it. It deserves, and should receive, the attention of some one competent to the task of unveiling its mysteries, and pointing out the means by which it may be disarmed of its malignity; and the author will feel fully repaid for his labour if it will, in the slightest degree, prove the means of directing more general attention to this hitherto much neglected disease.—*American Journal of Medical Sciences.*

---

*Excision of the Elbow Joint, in a case of Suppuration, and Caries of the Bones, by* Gurdon Buck, M. D., *one of the Surgeons of New York Hospital.*—I am not aware that another case of this operation occurring in this country, has been made public, except the one by Dr. Warren, of Boston, communicated by him verbally to Prof. Velpeau, of Paris, and alluded to in the second edition of his Mêdecine Operatorie, under the article "Excision of the Elbow Joint." Prejudice, or some other reason, appears to have deterred American Surgeons from resorting to it, notwithstanding the strong testimony in its favor, particularly in England and Scotland. A desire to confirm this testimony, and secure to this valuable operation the favor it deserves, and thus rescue some fellow creature from the deformity of an amputated limb, induces me to offer the case, incomplete as it is, with such details of its progress, and after treatment, as will be of service to those who may have occasion to repeat it.

John Wharton, a seaman, native of England, aged 25 years, with sandy hair, and fair complexion, was born of healthy parents, and had always enjoyed robust health, till within the last two or three years, when he had a severe attack of fever at sea in the month of July, soon after leaving a southern port. Several of his shipmates sickened and died of the same disease. While suffering from it himself, he was much exposed to wet and cold. Since that attack he has occasionally had pains in his back. On the 10th of last June, he was admitted to the hospital with inflammation of the right elbow. This commenced spontaneously six months before, with pain in the joint, and was followed in about six weeks, by a slight stiffness, which prevented complete flexion and extension, but still allowed him to use his arm and continue his usual occupation, until within two months prior to his admission. During these two months, which he passed on his voyage from Amsterdam, he was also disabled by lameness in the left hip. The condition of his arm, when admitted, was as follows:—It was kept in an extended position, the joint stiff and painful on attempting motion. There was increased heat and swelling about the elbow, with œdema of the forearm and hand. The arm was gradually brought to a right angle and supported in a sling. Cupping was directed every three days, and after several repetitions, blisters were resorted to. This plan of treatment was persevered in, without benefit, till the 13th of July, when the swelling had somewhat increased, and distinct fluctuation was perceptible over the external condyle. He suffered but little pain except on taking a deep inspiration, when he felt acute pain extending from the joint down the forearm. The greatest degree of swelling existed over the condyles and olecranon. Encouraged by previous success in similar cases, I now tried the actual cautery, and applied it five times over the posterior surface of the joint. On the separation of the eschars copious suppuration was established and kept up by appropriate dressings. August 11. An opening formed over the outer condyle and discharged a large collection of thin yellowish-white matter; ordered poultices. October 8. An extensive collection of matter had formed above the elbow, on the posterior and inner surface of the arm, covering its lower third; the parts around were œdematous, and doughy; with increased heat, and shining appearance of the skin. It was punctured, and discharged a large quantity of thin ill-conditioned fluid, mixed with lumps of curdy matter. Up to the 16th of January last, no improvement took place, notwithstanding various methods of treatment were resorted to; on the contrary, the disease of the elbow had extended, and the patient's general health had been affected by the constant local irritation. He had suffered at times from febrile paroxisms coming on with chills, and attended with profuse perspiration, and acute pain in the back at the lumbar region, that extended to the right side along the crest of the ilium, the character of which was obscure. His general strength had been pretty well sustained, and with few exceptions he had been able to go about. The organs of the chest were healthy. It was now obvious that no further delay was admissible, and recourse must be had to amputation or excision. The condition of the limb at this time was as follows:—It was kept at a right angle in a sling, and admitted only a slight degree of motion at the joint; the swelling, though very considerable, was confined to the immediate vicinity of the elbow, above and below which, the limb was very much wasted from long inaction. The opening over the outer condyle, had ulcerated to the size of half a dollar; and at the bottom of it, the head of the radius lay exposed, and rotated with the pronation and supination of the hand; though covered with granulations, the edge of the bone could be felt rough, and in a state of caries. Along the inner margin of the joint, there were two small openings with swollen edges; one, an inch below the olecranon, at the bottom of which the probe encountered this bony process in a denuded state; the other, at the same distance above the inner condyle, did not communicate with the bone. The skin and subjacent tissues covering the posterior surface of the joint were thickened, and of a dusky reddish color; there was but little increased heat in the part, and very little pain. The discharge was abundant and mixed with synovia. He retained the power of rotating the hand, but could not clench his fist. There had been an improvement in his general condition for several days previous to the operation; his appetite was good; his tongue clean, and bowels regular. He slept well, and was able to be about the whole day, though his countenance was rather languid and his cheeks flushed; pulse 92 and weak. He has suffered considerably of late, with pain in the back and left hip, but is now much relieved.

*Operation.*—January 16th. A tournequet being first applied to the arm high up, the patient was laid on his left side, and the right arm supported, with the elbow elevated and hand depressed.

A transverse incision was first made across the triceps muscle at its insertion, with a straight bistoury introduced (its back turned towards the ulnar nerve) at nearly a finger's breadth above and on the radial side of the inner condyle, and carried down to the bone; the point of the bistoury being made to graze its posterior surface and emerge at the outer condyle, while the edge was directed obliquely downwards, so as to keep close to the surface of the olecranon process.

Two longitudinal incisions were made from the extremities of this transverse one, extending an inch and a half above the condyles; similar ones were made, in continuation, below them, all forming together the letter H. The superior flap was dissected up from the bone, but the inferior included only the skin and fascia. The olecranon process being freed from the muscles and ligaments inserted on either side of it, was sawed about two-thirds through, at an inch and a quarter from its extremity, with the common amputating saw, and the section of it completed with a chisel and mallet. The ulnar nerve was drawn to the inside, while the muscular and ligamentous attachments were dissected from the condyles close to the bone. The disease appearing to have extended above the external condyle, it became necessary to make this section oblique, so as to include one inch of the extremity of the bone on its outer edge, and only half an inch on its inner. Nearly half an inch was then sawed off from the head of the radius, after first protecting the soft parts by slipping over it a slitted band of muslin, that served as a retractor. The rough inequalities of the bone, as well as the cartilage covering the coronoid process were pared away with bone forceps. All the soft parts were infiltrated and thickened, and the joint itself coated with a morbid product of a gelatinous appearance. This condition embarrassed the operation, by obscuring the parts and rendering it difficult to detach the soft parts from the bones; a considerable portion of this morbid tissue was dissected out. All the articular surfaces were found to be denuded of cartilage, rough and very vascular; at the bottom of the concavity of the olecranon, ulceration had extended into the cells of the bone, and formed a cavity capable of holding a small pea. The roughness extended an inch above the outer condyle, as already noticed.

The newly divided bony surfaces, though very vascular, had a healthy appearance. The hemorrhage was moderate during the operation, and only a single ligature was applied. The edges of the transverse incision were brought together by two sutures, and those of the longitudinal incision on each side by as many more, one being introduced above, and the other below the intersection of the two incisions. No suture was admissible to the incision below the outer condyle, from the existence of the ulcer at this point. Introducing the sutures, it was noticed that the upper longitudinal incision on the inside extended to within a finger's breadth of an old sinus; it was therefore prolonged to the sinus. Five sutures in all were employed, and between them strips of adhesive plaster applied, over which dry lint was placed, covered with a compress spread with simple cerate, and secured by a figure of eight bandage. The operation was a very painful one, and occupied about thirty minutes exclusive of the dressing. The limb was placed on a pillow, in a position intermediate between extension and right angle. On removal to his ward, the patient took an anodyne draught containing tinc. opii. ʒj; that was repeated in two hours, and at evening was ordered an effervescing mixture every two hours.

Jan. 17. Patient has passed a comfortable night, and had considerable sleep; he is free from pain; his pulse is 120, and temperature natural. There has been a free oozing of bloody serous fluid from the wound through the dressings. I divided some of the turns of bandage over the elbow on account of swelling of the forearm, directed the dressing to be kept wet with spirit. lotion; at evening he was comfortable and free from pain in the back and hip, with which he suffered before the operation; pulse 108.

20th. Has slept but little, though free from pain and uneasiness. Dressed the wound for the first time, and found adhesion had taken place between the edges of all the incisions; the ulcer over the head of the radius had improved in appearance, and discharged a considerable quantity of pus; the old sinus at the upper extremity of the wonnd on the inside also discharged freely: the sutures were left undisturbed, and the dressings reapplied as follows:—the joint was enveloped in a compress split into four tails, and spread with simple cerate, and over it strips of muslin were loosely applied, after the manner of the many tailed bandage. The limb was then laid in a guttered tin splint, bent at a very obtuse angle, and well padded with cotton; the whole supported in an easy position on pillows; his pulse was 104. He was allowed a few oysters and one pint of porter.

After a severe attack of erysipelas which had entirely disappeared by the 9th of February, on the 19th the patient is able to sit up all day and walk about the ward. He suffers again from his old complaint, pain in the back over the region of the right kidney, extending around the right side to the abdomen; there is some tenderness on pressure, but no swelling or induration. Ordered cups to the part. Ulcer on the outside of the joint has diminished in size, and suppurates but little from the old sinus on the inside; a lemon colored viscid fluid is discharged in small quantities. The arm can be drawn up by the sling so as to bring the fingers to the mouth, and can be extended out to a large obtuse angle. Began using a machine adapted to produce gradual extension to a straight position.

March 8th. There has been a progressive improvement in the condition of the elbow since the last date. The swelling and thickening of the tissues have diminished so far that the joint may now be considered of its proper size; its form is tapering from the joint upwards and downwards, and when flexed, is rounded at the elbow instead of being sharp; along the track of the inner and transverse incisions, three or four small openings with swollen edges still exist, from which oozes a slight discharge of viscid fluid, the ulcer on the outside is reduced to half its former dimensions, and suppurates but little; the patient is not yet capable of flexing and extending the arm, though he is conscious of returning strength in the limb; he can grasp an object with some force with his fingers. The hand inclines to a state of prostration, and is susceptible of being rotated only within narrow limits, so as to describe about one-fourth of a circle of which the inner edge of the hand is supposed to form the centre, and its outer edge, the circumference; any attempt to increase the degree of supination causes pain along the outer edge of the radius at the junction of its inferior and middle third. In moving the elbow, no feeling of crepitus has at any time been perceptible. The treatment has consisted in friction of the entire limb with spirit; simple dressings to the elbow, with a roller bandage from the hand to the shoulder; together with the use of the machine alluded to above, to extend the limb to a straight position. His progress has been retarded for a fortnight past, by the pain in his back, obliging him to keep quiet in bed, and thus preventing the use of his limb. I hope hereafter to give the further result of this operation.—*N. Y. Med. & Surg. Jour.*

Great interest must attach at present to the results of excisions of the joints, and every case, whatever its termination, should be recorded, as an act of duty. When amputation was regarded as less serious in its nature, and involving only the loss of a limb, with but little danger to life, it constituted almost the sole resource of the surgeon in incurable affections of the joints. It was, at that time, very properly regarded as more prudent to sacrifice a limb by an operation both simple and safe, than to endanger life by excision of a joint, which must always be accompanied by great pain, and of which the results were doubtful. Since it has been shown, however, that by the latter operation a most useful limb may be preserved to the patient, and, above all, since it has been demonstrated that amputation is one of the most serious operations to which the surgeon can resort—that one-fourth of all the amputated die—it becomes all important to determine the average mortality after excision. Excision of a joint, when successful, preserves the patient from a dreadful mutilation, and should the danger to life be found to be no greater than after amputation, it should be unhesitatingly preferred in many cases of diseases of the joints. Should the average mortality of excision be found *less* than that of great amputations, it should supersede the latter in all cases where the disease was sufficiently limited in extent to render it applicable. In a word, the question of preference must be decided by the relative danger of the two operations, and this latter can only be determined by an analysis of cases, all of which should therefore be recorded.

The merit of introducing the operation into this country is, we believe, due to Dr. Thomas Harris, of this city, who excised an elbow at the Pennsylvania Hospital in 1836. The woman, who atributes her disease—caries of the extremities of the bones—to the kick of a cow, had been under treatment eight or nine months in the Hospital with but little benefit, her general health was failing, and an operation became indispensable: the choice was presented to her, and she decided in favour of excision. The operation of Symes was accordingly performed, and the patient left the hospital with the wound entirely healed, and with considerable motion in the part. At present, five years after the operation, she is possessed of nearly all the motions of the part, extension being the most limited: the wound has never re-opened, and she finds the arm almost as useful as the one of the opposite side.

We will probably present detailed notes of this case—which we believe have not yet been published—in a future number.

### HEALTH OF THE CITY.

INTERMENTS *in the City and Liberties of Philadelphia, from the 17th to the 24th of April,* 1841.

| Diseases. | Adults. | Children. |
|---|---|---|
| Abscess of liver, | 1 | 0 |
| Amentia, | 1 | 0 |
| Apoplexy, | 1 | 0 |
| Casualties, | 0 | 1 |
| Croup, | 0 | 3 |
| Congestion of the brain, | 1 | 0 |
| Childbed, | 1 | 0 |
| Consumption of the lungs, | 18 | 1 |
| Convulsions, | 0 | 5 |
| Dropsy, | 2 | 0 |
| —— heart, | 1 | 0 |
| —— abdominal, | 1 | 0 |
| —— head, | 0 | 2 |
| —— breast, | 3 | 1 |
| Disease of brain, | 2 | 1 |
| —— bronchi, | 1 | 0 |
| Debility, | 1 | 1 |
| Effusion on chest, | 1 | 0 |
| Erysipelas, | 0 | 1 |
| Fever, remittent, | 1 | 0 |
| —— puerperal, | 1 | 0 |
| —— scarlet, | 0 | 1 |
| Inflammation of the breast, | 0 | 2 |
| —— brain, | 2 | 3 |
| —— bronchi, | 0 | 7 |
| —— lungs, | 1 | 6 |
| —— stomach, | 1 | 0 |
| —— bowels, | 0 | 2 |
| —— kidneys, | 1 | 0 |
| —— heart, | 1 | 0 |
| —— peritonæum, | 1 | 0 |
| Intemperance and exposure, | 1 | 0 |
| Inanition, | 0 | 1 |
| *Carried forward,* | 45 | 38 |

| Diseases. | Adults. | Children. |
|---|---|---|
| *Brought forward,* | 45 | 38 |
| Jaundice, | 1 | 0 |
| Malignant sore throat, | 0 | 2 |
| Marasmus, | 0 | 4 |
| Malformation, | 0 | 1 |
| Measles, | 0 | 10 |
| Mania a potu, | 1 | 0 |
| Old age, | 3 | 0 |
| Palsy, | 2 | 0 |
| Pleurisy, | 1 | 0 |
| Salivation, | 1 | 0 |
| Small pox, | 3 | 3 |
| Still-born, | 0 | 12 |
| Stricture of œsophagus, | 0 | 1 |
| Syphilis, | 1 | 0 |
| Tetanus, | 0 | 1 |
| Teething, | 0 | 1 |
| Unknown, | 1 | 0 |
| Total, 132— | 59 | 73 |

Of the above, there

| | |
|---|---|
| were under 1 year | 27 |
| From 1 to 2 | 19 |
| 2 to 5 | 15 |
| 5 to 10 | 9 |
| 10 to 15 | 0 |
| 15 to 20 | 3 |
| 20 to 30 | 17 |
| 30 to 40 | 12 |
| 40 to 50 | 11 |
| 50 to 60 | 8 |
| 60 to 70 | 4 |
| 70 to 80 | 4 |
| 80 to 90 | 3 |
| Total, | 132 |

